# When the virus hits suddenly: COVID-19 mimicking a subarachnoid haemorrhage—a case report and concise review of the literature

**DOI:** 10.1093/omcr/omab133

**Published:** 2022-01-24

**Authors:** Martha Pretorius, Immo Weichert

**Affiliations:** Acute Medicine Department, Ipswich Hospital, East Suffolk and North Essex NHS Foundation Trust, Heath Road, Ipswich, Suffolk, IP4 5PD, UK; Acute Medicine Department, Ipswich Hospital, East Suffolk and North Essex NHS Foundation Trust, Heath Road, Ipswich, Suffolk, IP4 5PD, UK

## Abstract

We report a clinical case, where COVID-19 presented with a thunderclap headache and collapse, but no fever or respiratory symptoms on initial presentation. The patient was worked up for a possible spontaneous subarachnoid haemorrhage (SAH), but had a normal CT brain and normal lumbar puncture and then very rapidly deteriorated with worsening respiratory failure and COVID-19 pneumonitis. We discuss the current evidence of neurological involvement by SARS-COV-2 and the proposed pathophysiological mechanisms underlying these presentations.

## INTRODUCTION

Atypical symptoms, such as diarrhoea and headache, add complexity to the diagnosis and management of COVID-19. There is a risk of missing early presentations of COVID-19 in the absence of respiratory symptoms. This may lead to incorrect management of patients and the spread of disease to vulnerable inpatients and the community. In the current climate, clinicians may also run the risk contributing all presenting symptoms to COVID-19 disease and miss other important diagnoses.

## CASE REPORT

A 54-year-old man presented with a thunderclap headache and collapse in April 2020. He had been well when he went to bed but was woken in the early hours by a severe headache. It was immediately at a level of 9/10 and encompassed his whole head from the level of his zygomas upwards. He briefly collapsed, there were no abnormal movements, and he regained awareness quickly according to the collateral by his wife. He had mild dizziness and nausea but no vomiting or photophobia. He had not lost his sense of taste or smell. His headache was worse on sitting and standing but improved on lying down and did not respond to simple analgesia. It was associated with pain at the temporomandibular joint and stiffness of his neck muscles. There were no respiratory symptoms or other symptoms of an infection. He was normally fit and well with no history of headaches prior to this admission. His only medical history was of mild ulcerative colitis for which he was not taking medications. No other family members or direct contacts of the patient were ill. The patient had not travelled abroad. He was a non-smoker. He had no significant family history.

**Figure 1:**
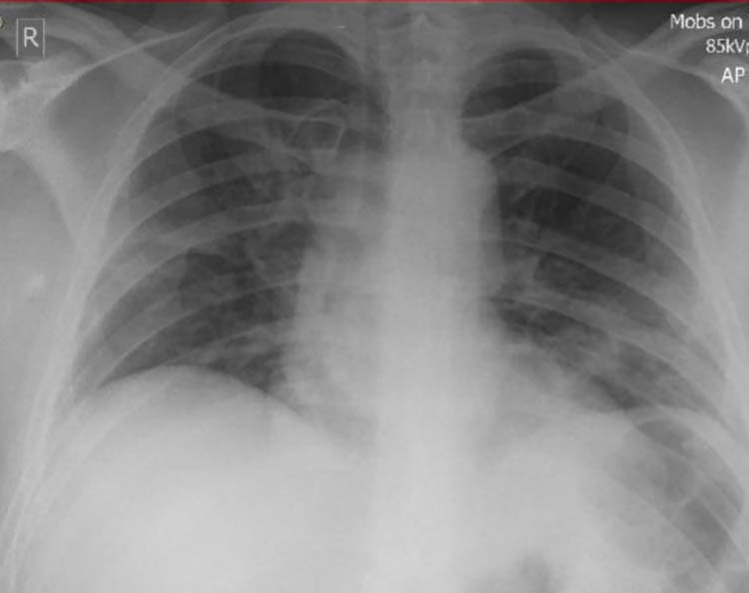
Chest X-Ray of patient on day 2 postadmission (Courtesy of East Suffolk North Essex NHS Foundation Trust Radiology Department).

The patient was seen by the GP who sent him by ambulance to the Emergency Department (ED) for a suspected spontaneous SAH. He underwent a CT brain scan, which was normal. The patient was then transferred to the medical admissions unit. On arrival, he had borderline pyrexia of 37.7°C and a tachycardia of 109 bpm. The rest of his observations and examination was unremarkable. A lumbar puncture was performed to rule out SAH or meningitis. The results of this are listed together with a timeline of events in Fig. 1 and Table 1.

**Table 1:** Timeline of the patient’s initial presentation and hospital stay

Day 0	03:00	Sudden onset headache 9/10 followed by collapse with associated nausea
		Patient took ibuprofen and paracetamol and attempted to rest but headache not resolving
Day 1	14:18	Woke up with persistent headache. Seen by the GP and referred to ED. GP office vitals: HR 68, BP 130/70, apyrexial
	16:16	Presented to the Emergency Department (ED) Green Zone—severe headache 7/10, worse on standing and Pain over TMJ. Sudden onset frontal to posterior headache associated with dizziness, gripping around the back of his head. Vitals: HR 109 BP 126/77 RR 15, low-grade fever of 37.7°C.
	17:31	CT head—No evidence of intracranial haemorrhage or collection. No major vascular abnormalities. No focal lesions. Normal CSF spaces. No bone deposits or fractures
	19:42	Transferred to the Acute Medicine Unit Green Zone
	20:46	Lumbar puncture performed, normal opening pressure, red blood cells = 26 cells 10*6/L, CSF protein = 0.28 g/L (reference range 0.15—0.40); glucose = 3.8 mmol/l; Gram stain showed no organisms, Xanthochromia negative, standard PCR meningitis panel shows no other viruses. No coronavirus PCR done on CSF as restrictions on testing were in place
Day 2	02:00	Fever recorded overnight (38.2°C)
	09:00	Peripheral oxygen saturations 91–92% on air with no shortness of breath or discomfort. COVID swab taken for PCR. Patient isolated Chest-X Ray showed bilateral basal airspace opacification suggestive of infection
Days 3–6		Slowly increasing oxygen demand with increasing discomfort and shortness of breath. Headache resolving
Day 7	11:30	Oxygen demand increased to 40% humidified O_2_^*^
	18:59	Further increase in oxygen demand to 60% humidified O_2_^*^
	20:11	Saturations 85% on 60% humidified O_2_ Increased to 15 litres non-rebreather mask. Reviewed by critical care outreach team
Day 8	03:43	Deteriorated further with saturations 91.7% on 15 litres non-rebreather mask.^*^ Intensive care review and subsequent transfer
	05:05	Oxygen saturations 89% on 15 l non-rebreather mask. Intubated
Day 10	06:00	Extubated
Day 11	15:00	Step down to ward
Day 15		Discharge from hospital

^*^For arterial blood gas see table.

He was kept in for observation and management of his ongoing headache. During the first night in hospital, he developed fever and hypoxia. The patient was isolated, a COVID-19 nasopharyngeal swab was taken, and a chest X-Ray performed. This showed bilateral lower zone infiltrates. He was treated with antibiotics in line with local antimicrobial guidelines. His nasopharyngeal swab returned subsequently positive for SARS-COV-2. There was no established COVID-19 treatment available at this point in the pandemic. He had not had any known COVID-19 contacts prior to admission.

Whilst the headache settled within the first 48 hours, the patient deteriorated over the next few days with worsening respiratory failure, developing COVID-19 according to the WHO case definition. On day 8, he was transferred to intensive care where he was intubated and ventilated before being able to return to the ward two days later after rapidly improvement (Tables 2 and 3). He made a full recovery and was discharged on day 15. There were no established treatments for COVID-19 at the time and patient received supportive care as per hospital guidelines.

**Table 2:** Blood results on admission and on day 7 before transfer to intensive care

	Day 1	Day 7	Reference range
Haemoglobin (g/L)	136	138	135—175
WCC (×10^9^/L)	5.0	6.3	4.0—11.0
Lymphocytes (×10^9^/L)	0.7	0.7	1.0—4.0
Neutrophils (×10^9^/L)	3.8	5.2	2.0—7.5
Platelets (×10^9^/L)	136	206	135—450
Urea (mmol/L)	4.5	6.4	2.5—7.8
Creatinine (mmol/L)	97	86	59—104
CRP (mg/L)	66	164	0—5
D-Dimer (ng/mL)	–	1085	0—500
Fibrinogen (g/L)	7.13 L	9.27	2—4.5

**Table 3:** Arterial blood gas analysis showing the rapid deterioration of the patient’s respiratory function around the time of being admitted to intensive care

Day of admission	7	7	8
Time	11:58	18:59	03:43
pH	7.501	7.49	7.502
PaCO_2_ (kPa)	4.89	4.71	4.68
PaO_2_ (kPa)	10.7	8.8	7.7
HCO_3_ (mmol/L)	29.0	27	28
Saturation (%)	96.5	94.2	91.7
FiO_2_ (%)	40%	60%	80%^*^

^*^via non-rebreather mask.

## DISCUSSION

The initial presentation of our patient with a thunderclap headache and collapse raised the suspicion of an aneurysmal SAH. The patient was managed accordingly. The presentation would also fit a diagnosis of viral meningitis. His CSF results are not in keeping with acute viral meningitis, but cases with normal cell counts and biochemistry have been reported in a systematic review [1]. The headache reported in our case is similar to that of a 15-year-old boy [2]—with many similar characteristics in terms of aggravating factors and description of symptoms. In this case, however, the headache was associated with raised intracranial pressure. Unfortunately, a CSF opening pressure was not performed in this case. It is important to note that the occurrence of SAH and COVID-19 may be incidental and unrelated to each other and the association of these is subject to further study.

Coronaviruses may cause a wide spectrum of neurological complications [3]. A recent survey of patients [4] has shown that the nature of headaches associated with SARS-COV-2 infection include a duration of more than 72 hours, bilateral nature and resistance to simple analgesia. These headaches also occur more commonly in men and are associated with nausea, phonophobia, photophobia, anosmia and ageusia.

These associated symptoms led to the theory that the olfactory bulb may act as entry point for SARS-COV-2, allowing direct infiltration into the central nervous system [5]. The biological basis for neurological COVID-19 disease may be dependent on several different biological mechanisms including ACE2-receptors in the cerebral vasculature and inflammatory infiltration via the blood–brain barrier [5]. Postmortem examination of three patients with severe SARS-COV-2 infection confirmed the presence of viral spike protein within the human brain [5].

Research has suggested that there is a link between initial neurological symptoms and the severity of subsequent respiratory complications in COVID-19 [5, 6]. Our case highlights that patients presenting with neurological symptoms prior to respiratory symptoms need close clinical monitoring as they may be at higher risk of more severe disease [6, 7]. Conversely, patients with a more severe clinical picture of COVID-19 were more likely to develop neurological manifestations, including acute cerebrovascular disease, haemorrhage and disturbance in level of consciousness [7]. A study of 224 inpatients in Wuhan found that neurological manifestations were significantly more common in severe infections (45%) when compared with less-severe infections (30.2%) [7]. Questions remain regarding the involvement of the brainstem in COVID-19 and its possible role in the development of respiratory failure.

Further research is needed to prove definitively that SARS-COV-2 can cause meningitis and encephalitis in humans, but case reports have shown that SARS-COV-2 RNA can be isolated in human CSF [8]. Severe manifestations of neurological disease in the form of acute necrotising encephalitis have been presented in a case report [9]. This disease process is linked to the cytokine storm phenomenon in severe viral disease. This is another different pathophysiological mechanism to be considered in addition to direct viral infiltration and vascular mechanisms as the cause of neurological disease in COVID-19 [9].

Cases of cerebral venous sinus thrombosis (CVST) associated with COVID-19 infection have been reported in case reports [10]. No MRI venogram was performed in this case as the history and symptoms were not pathognomonic with CVST, but this is something that should be considered in COVID-19 patients presenting with headache.

The spectrum of neurological disease in SARS-COV-2 infection is wide and varied, yet it is important to remember that not all headaches in patients with SARS-COV-2 infection are necessarily related to the disease and other diagnoses should still be considered [11].

## CONFLICT OF INTEREST STATEMENT

There is no conflict of interest to disclose

## FUNDING

This project received no specific grant from any funding agency in the public, commercial or not-for-profit sectors.

## ETHICAL APPROVAL

Patient information was de-identified and consent for publication has been obtained.

## CONSENT

This case study has been published with the written consent of the patient involved.

## GUARANTOR

Immo Weichert.
